# Syphilis Laboratory Guidelines: Performance Characteristics of Nontreponemal Antibody Tests

**DOI:** 10.1093/cid/ciaa306

**Published:** 2020-06-24

**Authors:** Susan Tuddenham, Samantha S Katz, Khalil G Ghanem

**Affiliations:** 1 Johns Hopkins University School of Medicine, Baltimore, Maryland, USA; 2 Centers for Disease Control and Prevention, Atlanta, Georgia, USA

**Keywords:** syphilis, *Treponema pallidum*, diagnostic testing, serologies

## Abstract

We reviewed the relevant syphilis diagnostic literature to address the following question: what are the performance characteristics, stratified by the stage of syphilis, for nontreponemal serologic tests? The database search included key terms related to syphilis and nontreponemal tests from 1960–2017, and for data related to the venereal disease research laboratory test from 1940–1960. Based on this review, we report the sensitivity and specificity for each stage of syphilis (primary, secondary, early latent, late latent, or unknown duration; tertiary as well as neurosyphilis, ocular syphilis, and otic syphilis). We also report on reactive nontreponemal tests in conditions other than syphilis, false negatives, and automated nontreponemal tests. Overall, many studies were limited by their sample size, lack of clearly documented clinical staging, and lack of well-defined gold standards. There is a need to better define the performance characteristics of nontreponemal tests, particularly in the late stages of syphilis, with clinically well-characterized samples. Published data are needed on automated nontreponemal tests. Evidence-based guidelines are needed for optimal prozone titrations. Finally, improved criteria and diagnostics for neurosyphilis (as well as ocular and otic syphilis) are needed.

Since *Treponema pallidum* cannot be cultured, and direct detection methods are not routinely available in most clinical settings, the detection of nonspecific or nontreponemal and treponemal antibodies forms the mainstay of syphilis laboratory diagnoses. Of note, the terms “nonspecific” or “nontreponemal” antibodies would be more accurately termed “antiphospholipid” antibodies, since they represent host antibodies made in response to phosphatidylcholine taken up from mammalian tissue by *T. pallidum*. However, these terms are commonly used in the literature and in clinical practice, so we have elected, for clarity, to use them in this document. For further discussion of treponemal-specific antibodies, please see Park et al in this issue. Antiphospholipid antibodies are used in combination with treponemal antibodies in the clinical context to help diagnose infections with *T. pallidum*. The primary antiphospholipid antibody tests in current use are the rapid plasma reagin (RPR), the venereal disease research laboratory (VDRL) and, to a much lesser extent, the toluidine red unheated serum test (TRUST) and unheated serum reagin. Both nonautomated and automated platforms are available to detect these antibodies. Nontreponemal tests are generally performed on serum, but some may also be performed on cerebrospinal fluid (CSF) to aid in the diagnosis of neurosyphilis. Finally, nontreponemal antibody titers are used to monitor treatment responses, although this is not the focus of this review [1].

The diagnosis of any syphilis stage relies on a clinical evaluation of patient symptoms and medical history, as well as on an interpretation of laboratory tests. As syphilis rates continue to rise throughout the United States, there is a need to systematically identify the performance characteristics of nontreponemal tests to aid laboratorians as they seek to provide support to clinicians for syphilis diagnoses. We sought to review the literature to address this question: what are the performance characteristics, stratified by the stage of syphilis, for nontreponemal serologic tests?

## METHODS

We searched Medline, Embase, the Cumulative Index to Nursing and Allied Health Literature database, Scopus, and the Cochrane Library from January 1960–June 2017 for the terms “syphilis” OR “*Treponema pallidum*” OR “neurosyphilis” AND “serodiagnosis” OR “serum” OR “plasma” OR “test” OR “exam” OR “assay” OR “screen” OR “laboratory” OR “diagnosis” OR “nontreponemal” OR “algorithm” OR “antibody titer” OR “serofast” OR “VDRL” OR “venereal disease research laboratory” OR “RPR” OR “rapid plasma reagin” OR “toluidine red unheated.” Animal studies and those not written in English were excluded. Conference abstracts were excluded. This search identified a total of 4452 documents after duplicate exclusions. We conducted an additional search for the terms “VDRL” OR “venereal disease research laboratory” from 1940–1960, and applied similar exclusions. This yielded an additional 82 documents.

The combined literature search resulted in 4534 documents. Abstracts for these 4534 publications were reviewed, papers not relevant to syphilis or to nontreponemal syphilis serologies were excluded, and 452 publications were selected for full review. Those that did not address the relevant topic, did not include primary data, related solely to the serofast state, or focused on tests not currently in use were excluded. This yielded 138 papers that were abstracted. A review of references of these 138 papers revealed an additional 2 relevant publications for a total of 140 papers. Additionally, data were obtained from the Food and Drug Administration (FDA) on the 2 approved, automated nontreponemal tests whose primary data had not yet been published.

Papers were assigned to several categories: (1) false negatives; (2) positive nontreponemal tests in conditions other than syphilis (including subsections for pregnancy, leprosy, illicit drug use, malaria, human immunodeficiency virus [HIV], hepatitis C virus, autoimmune diseases, endemic treponematoses, and vaccinations); (3) primary syphilis; (4) secondary syphilis; (5) early latent syphilis; (6) late latent syphilis; (7) tertiary syphilis; (8) neurosyphilis; (9) ocular syphilis; (10) otic syphilis; and (11) automated nontreponemal tests. Papers were additionally categorized by relevance and quality, into those which were most relevant and of high quality, those of moderate quality and relevance, and those of lower quality and relevance, based on specific criteria detailed separately in each syphilis category below. Several studies reported on composite endpoints (eg, primary and secondary syphilis together or “nonspecific” latent syphilis), which limited their impact. Data abstracted from these studies were not included in the findings below.

## RESULTS AND DISCUSSION

Overall, many of the studies reviewed were retrospective and had small sample sizes. Many were also limited by a lack of clearly documented clinical staging and well-defined gold standards. Documentation of the treatment status or the time interval between treatment and sample collection was not always clear. There were no published papers examining the performance characteristics of the 2 existing FDA-approved, automated nontreponemal assays at the time that the literature review was conducted. With these caveats, more detailed findings for each subgroup follow below.

### Primary Syphilis

For primary syphilis, we identified 13 high-quality papers (see Tables 1 and 2) [2–14] with a gold standard based on clinical diagnoses with a positive darkfield on primary lesions; 8 moderate-quality papers with a gold standard based on polymerase chain reaction (PCR) from lesions or a combination of a clinical diagnosis and serology [15–22]; and 9 lower-quality papers with a gold standard that was not well defined [23–31]. Based on high-quality papers, the sensitivity of VDRL in primary lesions ranged from 62.5–78.4%, although 1 high-quality paper reported a sensitivity of 50% based on 76 patients with darkfield-confirmed primary syphilis [12]. Based on high-quality papers, the sensitivity of the RPR ranged from 62.5–76.1%, although 1 high-quality paper (Huber et al [10]) reported a sensitivity of 92.7% based on 109 patients with darkfield-confirmed primary syphilis. Another high-quality paper reported a sensitivity for the reagin screening test of 57.7% [7]. Amongst the papers reporting both RPR and VDRL results, the serum RPR was generally as or slightly more sensitive than the VDRL [2–4, 7, 8, 10]. Few studies examined serum nontreponemal test specificity in the setting of primary syphilis; however, 1 moderate-quality study (Ballard et al [15]; see Table 1) enrolled 868 patients with genital ulcer disease and conducted *T. pallidum* real-time PCR testing on them. They found that the RPR had specificities for syphilis of 90.6% in people living with HIV and 87.3% in those living without HIV in the setting of genital ulcer disease [15].

**Table 1.** Summary of the Relevant Data

Study, Authors, Year [Ref]	Study Design	Study Population	Gold Standard	Findings
Primary syphilis				
Creegan et al, 2007 [3]	Retrospective cross-sectional	n = 106 with primary syphilis. 31% HIV+	DF microscopy	Sensitivity: VDRL+: 77/106 = 72.6% RPR+: 37/51 = 72.5%
Bossak et al, 1960 [2]	Retrospective cross-sectional	n = 119 with primary syphilis	DF microscopy	Sensitivity: VDRL+: 79/119 = 66.4% USR+: 85/119 = 71.4% RPR-US+: 86/119 = 72.3%
Dyckman et al, 1976 [7]	Cross-sectional	n = 111 primary syphilis	DF microscopy, no symptoms of secondary syphilis	Sensitivity: VDRL+: 70/111 = 63.1% RPR+: 72/111 = 64.8% RST+: 64/111 = 57.7%
Dyckman et al, 1978 [4]	Cross-sectional	n = 80 with primary syphilis	DF microscopy, no symptoms of secondary syphilis	Sensitivity: VDRL+: 50/80 = 62.5% RPR+: 50/80 = 62.5%
Dyckman et al, 1980 [6]	Cross- sectional	n = 63 with primary syphilis	DF microscopy, no symptoms of secondary syphilis	Sensitivity: VDRL+: 48/63 = 76.2% RST + without anticoagulant: 49/63 = 77.8% RST + with EDTA: 50/63 = 79.4% RST + with citrate: 50/63 = 79.4% RST + with heparin: 49/63 = 77.8%
Dyckman et al, 1980 [5]	Cross-sectional	n = 130 with primary syphilis	DF microscopy	Sensitivity: VDRL+: 89/130 = 68.5%
Falcone et al, 1964 [8]	Cross-sectional	n = 134 with primary syphilis	DF microscopy	Sensitivity: VDRL+: 105/134 = 78.4% RPR+: 102/134 = 76.1%
Greaves, 1962 [9]	Cross-sectional	n = 13 with primary syphilis	DF microscopy	Sensitivity: VDRL+: 10/13 = 76.9%
Huber et al, 1983 [10]	Cross-sectional	n = 109 with primary syphilis	DF microscopy	Sensitivity: VDRL+: 79/109 = 72.5% RPR+: 101/109 = 92.7%
Lassus et al, 1967 [11]	Cross-sectional	n = 62 with primary syphilis	DF microscopy	Sensitivity: VDRL+ = 63%
Moore and Knox, 1965 [12]	Retrospective cross-sectional	n = 76 with primary syphilis	DF microscopy	Sensitivity: VDRL+ = 50%
Wende et al, 1971 [13]	Retrospective cross-sectional	n = 322 with primary syphilis	DF microscopy	Sensitivity: VDRL+: 236/322 = 73.3%
Sischy et al, 1991 [14]	Cross-sectional	n = 21 with primary syphilis	DF microscopy	Sensitivity: RPR+: 15/21 = 71%
Ballard et al, 2007 [15]	Cross-sectional	n = 868 patients with GUD enrolled in South Africa.	Multiplex PCR for *T. pallidum*, *H. ducreyi*, and HSV	*T. pallidum* was detected in 163 patients by real-time PCR Sensitivity of RPR HIV+: 81.8% Specificity of RPR HIV+: 90.6% Sensitivity of RPR HIV−: 78.6% Specificity of RPR HIV−: 87.3%
Secondary syphilis				
Moore and Knox, 1965 [12]	Retrospective cross-sectional	n = 100 with secondary syphilis	DF microscopy	Sensitivity: VDRL+ = 100% RPR+ = 91%
Bossak et al, 1960 [2]	Retrospective cross-sectional	n = 98 with secondary syphilis	DF microscopy	Sensitivity: VDRL+: 98/98 = 100% USR+: 98/98 = 100% RPR-US+: 98/98 = 100%
Castro et al, 2003 [16]	Prospective cross-sectional study	n = 25 with secondary syphilis	FTA-ABS+ and clinical findings	Secondary syphilis: RPR+: 25/25 = 100%
Dyckman et al, 1976 [7]	Cross-sectional study	n = 56 with secondary syphilis	DF + secondary lesions OR 2 or more symptoms of secondary syphilis	Sensitivity: VDRL+: 56/56 = 100% RPR+: 56/56 = 100% RST+: 56/56 = 100%
Dyckman et al, 1978 [4]	Cross-sectional study	n = 29 with secondary syphilis	DF + secondary lesions OR 2 or more symptoms of secondary syphilis	Sensitivity Secondary syphilis: VDRL+: 29/29 = 100% RPR+: 29/29 = 100% SyphlaCheck+: 29/29 = 100%
Dyckman and Wende, 1980 [6]	Cross-sectional study	n = 23 with secondary syphilis	DF + secondary lesions OR 2 or more symptoms of secondary syphilis	Sensitivity: VDRL+: 23/23 = 100% RST + without anticoagulant 23/23 = 100% RST + with EDTA: 23/23 = 100% RST + with citrate: 23/23 = 100% RST + with heparin: 23/23 = 100%
Falcone et al, 1964 [8]	Cross-sectional study	n = 217 with secondary syphilis	DF microscopy	Sensitivity: RPR+: 211/217 = 97.2% VDRL+: 217/217 = 100%
Greaves, 1962 [9]	Cross-sectional study	n = 16 with secondary syphilis	DF microscopy	Sensitivity: VDRL+: 16/16 = 100%
Gibowski et al, 1998 [17]	Cross-sectional study	n = 17 with recent secondary syphilis; n = 44 with recurrent secondary syphilis	Clinical staging and + FTA-ABS, TPHA, Captia Syphilis M	Sensitivity: VDRL+ Recent secondary [17] = 100% Recurrent secondary [44] = 100%
Glicksman et al, 1967 [18]	Cross-sectional study	n = 31 with secondary syphilis	Clinical staging and + VDRL	Sensitivity: RPR+ = 100%
McMillan and Young, 2008 [20]	Prospective study	n = 68 with secondary syphilis	Clinical staging and treponemal tests	Sensitivity: VDRL+ = 100%
Early latent				
Gibowski et al, 1998 [17]	Cross-sectional study	n = 34 with early latent syphilis	Clinical staging and + FTA-ABS, TPHA, Captia Syphilis M	Sensitivity: VDRL+ = 100%
McMillan and Young, 2008 [20]	Prospective	n = 72 with early latent syphilis	Infection within 2 years, clinical staging, and treponemal tests	Sensitivity: VDRL+ = 85%
de Lemos et al, 2007 [27]	Retrospective cross-sectional	n = 23 with early latent syphilis	Clinical, lab (FTA-ABS, TPHA, VDRL) and epidemiologic criteria not further defined	Sensitivity: VDRL+: 23/28 = 82.1%
Backhouse and Nesteroff, 2001 [23]	Retrospective cross-sectional	n = 6 with early latent syphilis	Clinical and serologic not further defined	Sensitivity: VDRL+: 6/6 = 100%
Late latent				
Gibowski et al, 1998 [17]	Cross-sectional	n = 44 with late latent syphilis	Clinical staging + FTA-ABS, TPHA, Captia Syphilis M	Sensitivity: VDRL = 63.6%
Singh et al, 2008 [21]	Retrospective case series	n = 1303 with late latent syphilis	FTA-ABS or MHA-TP, and clinical context	Sensitivity: RPR+: 791/1303 = 61%
de Lemos et al, 2007 [27]	Retrospective cross-sectional	n = 44 with late latent syphilis	Clinical, lab (FTA-ABS, TPHA, VDRL) and epidemiologic criteria not further defined	Sensitivity: VDRL+: 29/44 = 65.9%
Backhouse and Nesteroff, 2001 [23]	Retrospective cross-sectional	n = 12 with late latent syphilis	Clinical and serologic not further defined	Sensitivity: VDRL+: 9/12 = 75%
Tertiary				
de Lemos et al, 2007 [27]	Retrospective cross-sectional	n = 17 with tertiary syphilis	Clinical, lab (FTA-ABS, TPHA, VDRL) and epidemiologic criteria not further defined	Sensitivity: VDRL+: 8/17 = 47%
Thakar et al, 1996 [31]	Cross-sectional study	n = 58 with tertiary syphilis	“Clinically suspected” not otherwise defined	Sensitivity: VDRL+: 37/58 = 63.8%
Neurosyphilis				
Castro et al, 2008 [32]	Retrospective cross-sectional	Cases: n = 25 with NS (8 were SNS and 24 were HIV+) Controls: n = 163 with + syphilis serologies but no e/o NS; n = 126 with no syphilis but with other neurologic disorders	Positive serum serologic test for syphilis, reactive CSF FTA-ABS, increased CSF protein ≥45 mg/dL, and CSF pleocytosis ≥10cell/mm^3^	Of the controls, 1 CSF VDRL and RPR were positive. CSF VDRL+: Specificity: 99% overall (ANS and SNS) Sensitivity: 70.8% Symptomatic NS (*n* = 8): 7/8 Sensitivity: 87.5% Asymptomatic NS (*n* = 16): 10/16 Sensitivity: 62.5% CSF RPR+: Specificity: 99.3% overall (ANS and SNS) Sensitivity: 75% Symptomatic NS (*n* = 8): 8/8 Sensitivity: 100% Asymptomatic NS (*n* = 16): 11/16 Sensitivity: 68.8%
Marra et al, 2017 [33]	Retrospective case study	Training data set (n = 191; 45 with *T. pallidum* PCR, 40 had symptoms). Validation data set (n = 380; 41 with *T. pallidum* PCR, 95 had symptoms)	(1) CSF VDRL positive OR (2) CSF PCR detection of *T. pallidum* OR (3) New vision or hearing loss (with clinical or serologic evidence of syphilis)	Validation data set: more were previously treated, far fewer had a positive CSF VDRL, and far fewer were *T. pallidum* PCR positive. *Training data set* Sensitivity of CSF VDRL+: (1) comp with PCR = 48.9% (34.3–63.5) (2) comp with symptoms = 67.5% (53–82) Specificity of CSF VDRL+: (1) comp with PCR = 74.0% (66.9–81.1) (2) comp with symptoms = 78.2% (71.4–85) *Validation data set* Specificity of CSF VDRL+: (1) comp with PCR = 91.2% (88.1–94.2) (2) comp with symptoms = 90.2% (86.7–93.6) No difference in sensitivity or specificity based on HIV status.
Marra et al, 2012 [34]	Retrospective cross-sectional study	n = 149, 39 with “lab defined NS” and 33 with “symptomatic NS.” Referred to study: neurologic findings, serum RPR > 1:32 and, in HIV+, CD4 ≤ 350.	(1) “Lab defined”: positive CSF FTA and CSF WBC > 20 (2) “Symptomatic”: vision or hearing loss (with clinical or serologic evidence of NS) RPRV technique: modified to be more similar to CSF VDRL technique, to adjust for lower concentration of immunoglobin in CSF vs serum	“Lab defined”: Sensitivity: CSF-VDRL+ = 71.8 (57.7–85.9) CSF-RPR+ = 56.4% (40.8–72.0) CSF-RPRV+ = 59.0% (43.6–74.4) Specificity: CSF-VDRL+ = 98.3% (95–100) CSF-RPR+ = 100% (100–100) CSF-RPRV+ = 98.3% (95–100) “Symptomatic”: Sensitivity: CSF-VDRL+ = 66.7% (50.6–82.8) CSF-RPR+ = 51.5% (34.4–68.6) CSF-RPRV+ = 57.6% (40.7–74.5) Specificity: CSF-VDRL+ = 80.2% (72.9–87.5) CSF-RPR+ = 89.7% (84.2–95.2) CSF-RPRV+ = 84.5% (77.9–91.1)
Zhu et al, 2014 [35]	Prospective cross-sectional	n = 210 NS patients, 56 asymptomatic, 154 symptomatic	Positive serum serologies, “Symptomatic”: clinical signs and symptoms with a positive CSF TPPA in absence of blood contamination. “Asymptomatic”: CSF WBC > −10 AND positive CSF TPPA in absence of blood contamination.	Combined for symptomatic NS and asymptomatic NS Sensitivity: VDRL+ = 81.4% (75.4–87.4) RPR+ = 76.2% (70.2–82.2) RPR-V+ = 79.5% (73.5–85.5) TRUST+ = 76.2% (70.2–82.2) Specificity: VDRL+ = 90.3% (88.3–92.3) RPR+ = 93.4% (91.4–95.4) RPRV+ = 92.7% (90.7–94.7) TRUST+ = 93.1% (91.1–95.1) Symptomatic NS: Sensitivity: VDRL+ = 85.7% (79.7–91.7) RPR+ = 81.8% (75.8–87.8) RPRV+ = 83.1% (77.1–89.1) TRUST+ = 82.5% (76.5–88.5) Specificity: VDRL+ = 86.7% (84.7–88.7) RPR+ = 90.2% (88.2–92.2) RPRV+ = 89.1% (87.1–91.1) TRUST+ = 90.1% (88.1–92.1) Asymptomatic NS: Sensitivity: VDRL+ = 69.6% (59.6–79.6) RPR+ = 60.7% (50.7–70.7) RPRV+ = 69.6% (59.6–79.6) TRUST+ = 58.9% (48.9–68.9) Specificity: VDRL+ = 79.4% (77.4–81.4) RPR+ = 82.6% (80.6–84.6) RPRV+ = 81.8% (79.8–83.8) TRUST+ = 82.1% (80.1–84.1)
Delaney, 1976 [36]	Case report	n = 1 with spinal cord tumor	Serum FTA-ABS and VDRL	In this patient, CSF was reactive to VDRL and FTA-ABS tests, but became nonreactive with removal of the spinal cord tumor. Serum serologies were persistently negative.
Izzat et al, 1971 [37]	Laboratory experiment	N/A	A laboratory experiment in which syphilitic blood was added to CSF to find at what titer and what amount it had to be added to get a positive CSF VDRL.	50 lambda for 1:1 titer blood and 3 lambda for 1:256 titer blood was necessary to induce a positive CSF VDRL. 3 lambda of whole blood per mL of CSF caused a definite blood color, whereas 1 lambda did not. With VDRL titers of 1:256 or below, sufficient whole blood to cause a false positive CSF VDRL test produces visibly bloody CSF.
Madiedo et al, 1980 [38]	Case report	n = 1	Serum FTA and VDRL	This was a patient meningeal carcinomatosis. Serum FTA and VDRL were negative, but CSF VDRL was positive and CSF FTA was negative. 1 week later both VDRL and FTA were positive in the CSF (though blood stayed negative)
Ocular syphilis				
Spoor et al, 1987 [39]	Retrospective case series	n = 50 with clinically defined ocular syphilis	Ocular findings and positive serum FTA-ABS	Sensitivity: Serum VDRL = 24% CSF VDRL = 0%
Tuddenham et al, 2015 [40]	Retrospective case series	n = 48 with positive serum treponemal and negative nontreponemal tests who had a lumbar puncture	Neurosyphilis defined by clinician judgement, as well as “definite” by positive CSF VDRL and “suspected” by CSF WBC > 5 and CSF protein > 50	Of 48 serodiscordant patients with an LP, only 2 were treated for neurosyphilis, and this diagnosis was doubtful even in these patients. Of 48 serodiscordant patients with an LP, 2 were treated for ocular syphilis and responded to treatment. Neither had a positive CSF VDRL. Neurosyphilis seems rare with a negative serum nontreponemal test, but ocular syphilis may be more likely to occur in this setting.
Kunkel et al, 2009 [41]	Retrospective case series	n = 24 with ocular syphilis, (11 HIV+, 13 HIV−)	(1) inflammatory disease of the eye, the optic nerve or orbital tissue; AND (2) serological evidence for syphilis (positive TPPA or TPHA with either positive VDRL > 1:4 or positive FTA-ABS; AND (3) improvement following adequate antimicrobial therapy.	Sensitivity: CSF VDRL+: 7/23 = 30.4% CSF VDRL+: 3/13 HIV− CSF VDRL+: 4/10 HIV+
Parc et al, 2007 [42]	Retrospective case series	n = 10 with syphilitic uveitis 8/10 HIV+	Active uveitis and positive serum MHA-TP	Sensitivity: CSF VDRL+: 2/9 = 22.2% Serum VDRL+: 10/10 = 100%
Ormerod et al, 2001 [43]	Retrospective case series	n = 21 with syphilitic posterior uveitis 5/21 HIV+	Positive serum RPR AND FTA AND “evidence of the appropriate pattern of active ocular inflammation”	Sensitivity CSF VDRL+: 7/19 = 36.8% “acute” (sx began within last 3 months) posterior uveitis: 5/8 “chronic” posterior uveitis (sx longer than 3 months): 2/11 that had test done, 18%
Browning, 2000 [44]	Retrospective case series	n = 14 patients with ocular syphilis 5/14 HIV+	Active disease of the vitreous, retina, retinal pigment epithelium, choroid, or optic nerve AND a positive FTA or MHA-TP.	Sensitivity CSF VDRL: CSF VDRL+: 2/9 that had testing = 22.2% Sensitivity serum RPR: Serum RPR+: 12/14 = 85.7%
Bollemeijer et al, 2016 [45]	Retrospective case series	n = 85 with syphilitic uveitis 28/85 HIV+	Positive TPPA or TPHA and/or positive FTA AND “agreement on the diagnosis of syphilitic uveitis between ophthalmologist, dermatologist, ID specialist and neurologist.”	Sensitivity: CSF VDRL+: 12/31 = 38.7% Serum VDRL+: 69/85 = 91.2%
Villaneuva et al, 2000 [46]	Retrospective case series	n = 20 with syphilitic posterior uveitis 3/9 tested were HIV+	Posterior uveitis and positive serum treponemal test	Sensitivity: CSF VDRL+: 2/15 = 13.3% Serum RPR+: 17/20 = 85%
Li et al, 2011 [47]	Retrospective case series	n = 13 with active posterior syphilitic uveitis. 10/12 people living with HIV	serum RPR AND treponemal test AND a clinical diagnosis of ocular syphilis with posterior segment findings (eg, retinitis choroiditis) attributable to syphilis.	8 patients had LP Sensitivity: CSF VDRL+: 2/8 = 25% positive None were serum RPR negative, but this was part of the diagnosis.
Restivo et al, 2013 [48]	Retrospective case series	n = 14 with ocular syphilis 11/14 had early syphilis; 6 were HIV+	Serological evidence of syphilis AND exclusion of other causes for ocular findings.	Sensitivity: CSF VDRL+: 3/7 (1 HIV + 2 HIV−) = 42.9%
Dai et al, 2016 [49]	Retrospective case series	n = 25 with ocular syphilis (HIV−)	Serological evidence of syphilis + ocular manifestations + CSF exam.	Sensitivity: CSF VDRL+: 9/25 = 36%
Kim et al, 2016 [50]	Retrospective series	n = 39 with ocular syphilis, 45 eyes	Serological evidence of syphilis + ocular manifestations	Sensitivity: Serum VDRL or RPR+: 32/39 = 82% CSF VDRL+: 0/6 = 0%
Lee et al, 2015 [51]	Retrospective case series	n = 16 with ocular syphilis (29 eyes [10 HIV+])	Serological evidence of syphilis AND ocular manifestations	Sensitivity: CSF VDRL+: 2/9 HIV+ = 22.2% CSF VDRL+: 1/6 HIV− = 16.7%
Shen et al, 2015 [52]	Retrospective case series	n = 13 with ocular syphilis (21 eyes [1 HIV+])	Serological evidence of syphilis + ocular inflammation	Sensitivity: Serum RPR+: 12/13 = 92.3% CSF RPR+: 3/11 = 27.2%
Mathew et al, 2014 [53]	Prospective study	n = 41 with ocular syphilis (63 eyes); 13 HIV+	Serological evidence of syphilis (both treponemal and nontreponemal reactive) + ocular inflammation + early syphilis stage	All had RPR/VDRL titers > 1:16 (part of definition to have positive nontreponemal serum serologies). HIV + median VDRL titer 1:256 HIV− median VDRL titer 1:128 (not statistically significant)
Rodrigues et al, 2014 [54]	Retrospective case series	n = 11 with ocular syphilis; 19 eyes (3 HIV+)	Serological evidence of syphilis + ocular inflammation	Sensitivity: Serum VDRL+: 8/11 = 72.7% CSF VDRL+: 2/9 = 22.2%
Yap et al, 2014 [55]	Retrospective case series	n = 12 with ocular syphilis; 18 eyes (8 HIV+)	Serological evidence of syphilis + active uveitis	Sensitivity: Serum RPR+: 10/12 = 91.7% CSF VDRL+: 3/6 = 50%
Puech et al, 2010 [56]	Retrospective case series	n = 8 with ocular syphilis (5/8 HIV+)	Serological evidence of syphilis + ocular manifestations	Sensitivity: Serum VDRL+: 8/8 (but part of inclusion criteria) CSF VDRL+: 1/5 = 20%
Otosyphilis				
Hughes and Rutherford, 1986 [57]	Prospective study	n = 5349, n = 25 found to have otologic syphilis	Otosyphilis: (1) active inner ear dysfunction not explained by other causes, with or without evidence for systemic syphilis, and (2) positive FTA-ABS serology with or without positive RPR serology. Control: (1) no evidence by history or physical examination of previous or present syphilis, and (2) a presenting complaint of hearing loss characterized by bilateral symmetric sensorineural hearing loss and age consistent with a diagnosis of presbycusis.	Sensitivity of serum RPR based on prevalence of 570/100 000: 55% Specificity of serum RPR based on prevalence of 570/100 000: 97%
Abuzeid and Ruckenstein, 2008 [58]	Retrospective case series	n = 181 with idiopathic progressive SNHL; n = 9 with otosyphilis	Positive TPA and SNHL	Sensitivity: Serum RPR+: 8/9 = 88.9%
Gleich et al, 1992 [59]	Retrospective case series	n = 18 with otosyphilis	Positive serum FTA and SNHL, tinnitus, or vertigo WITH a normal brainstem auditory response or posterior fossa MRI	Sensitivity: CSF VDRL+: 1/18 = 5.6% Serum VDRL+: 4/18 = 22.2%
Yimtae et al, 2007 [60]	Retrospective case series	n = 85 with otosyphilis	Positive serum VDRL AND treponemal test WITH cochleovestibular symptoms	Sensitivity: CSF VDRL+: 2/37 = 5.4%
False negatives				
el-Zaatari et al, 1994 [61]	Cross-sectional	n = 4328 sera tested by RPR and then rechecked with serial dilutions up to 16-fold. n = 2065 pregnant women; n = 1439 nonpregnant women; n = 824 men	FTA-ABS for false positives. Dilutions for prozone	Only 1 prozone reaction was detected in a man (overall prozone 95% CI was 0–.4%). Female nonpregnant BFP: 1.1% Female pregnant BFP: 0.6% Male BFP: 1.3%
el-Zaatari and Martens, 1994 [62]	Case report	“Case” report: 2 labs screening n = 2232 sera for syphilis with RPR, additional n = 1022 sera	N/A	1 lab reported 64/1210 (5.3%) as positive, the other lab reported 78/1210 (6.4%) as positive. The discrepancy was felt to relate to a cold centrifuge. When the temperature was adjusted from 4 to 27°C, an additional 1022 samples tested were consistent between the 2 laboratories.
Liu et al, 2014 [63]	Retrospective cross-sectional	n = 46 856 sera tested with RPR and TPPA. n = 1573 discrepant (RPR−, TPPA+) were diluted from 1:1 to 1:32.	TPPA, CIA, then dilution	Overall incidence of prozone phenomenon of .83% 36/1573 RPR−, TPPA + were prozone. Prozone reaction was most common in primary and secondary syphilis; neurosyphilis and pregnancy also increased the odds of prozone. Nearly 31% of patients titers with the prozone reaction were ≤1:16.
Causes of positive VDRL/RPR tests other than syphilis: General				
Sischy et al, 1991 [14]	Cross-sectional study	n = 1170 men with acute urethritis or genital ulceration who had physical exam, serum RPR, and FTA	BFP: Positive RPR and negative FTA with “no other signs or symptoms of syphilis”	RPR: 178/1149 without primary syphilis were positive. 2 of these were felt to be BFPs (they report as .02%).
Walker, 1971 [64]	Cross-sectional study	n = 6225 sera tested with RPR. If RPR+, was tested with VDRL, FTA, and TPI	BFP: FTA and TPI	RPR: 95/6225 tests done were BFPs = .015% 86 BFPs with VDRL (however, VDRL was tested only if RPR was positive)
Omer et al, 1982 [65]	Cross-sectional retrospective	n = 2201 blood donors and n = 199 with STDs tested with VDRL and FTA	BFP: VDRL + and FTA−	VDRL: 30/2201 BFPs in blood donor group = 1.36% overall prevalence BFP VDRL: 10/199 BFP in STD group = 5% overall prevalence
Liu et al, 2014 [66]	Retrospective study	n = 63 765 blood samples tested with RPR.	BFP: TPPA and CIA	RPR: 206 (0.32%) BFP. In multivariate analysis, an increased likelihood of the CBFP reaction was associated with female subjects, subjects ≥80 years old, and subjects between 16 and 35 years old.
Johansson et al, 1970 [67]	Cross-sectional study	n = 6737 dermatological inpatients tested with VDRL.	BFP: FTA-ABS	VDRL: 28/6737 BFP = .004%
Geusau et al, 2005 [68]	Retrospective study	n = 300 000 sera with age, sex, and stage of disease.	BFP: TPHA	VDRL: 2799 patients (.92%) of the study population were positive, of whom 736 (26%) were BFP. BFP reactivity was found in .24% and was higher in women than in men (.27% versus .20%; *P* < .001) and in patients over 60 years of age (.34%), as compared with those under 60 (.25%; *P* < .001). People living with HIV (n = 1415) had a 10-fold higher rate of BFP tests (2.1% versus .24)
Bala et al, 2012 [69]	Retrospective study	n = 5785 sera	BFP: TPHA Comparator: VDRL	VDRL: 68/80 had <1:8 titer on quantitation, TPHA was positive in 59 samples: BFP .2%. There were no BFPs among sera with VDRL titers of ≥1:8. The male-to-female ratio of BFP reactions was 2:1
Wiwanitkit, 2002 [70]	Prospective study	n = 30 with BFP, tested with VDRL every 2 weeks	BFP: TPHA	VDRL: Seroreversion occurred between 9.25 and 10.49 weeks; 25 returned to nonreactive by 10 weeks; 2 cases within 14 weeks.
Tuffanelli, 1966 [71]	Cross-sectional	n = 58 aged persons randomly selected from a Jewish old-age home.	BFP: FTA-ABS	RPR: 6/58 (9%) had persistently positive RPR for syphilis but negative FTA.
Smikle et al, 1990 [72]	Cross-sectional	n = 19 067 sera screened with VDRL. n = 441 general population, n = 145 pregnant women VDRL + with a titer <1:8 were confirmed with FTA	BFP: VDRL <1:8 with a negative FTA	General population: 94/347 + VDRL with titer <1:8 (27%) were BFP Pregnant women: 22/71 + VDRL with titer <1:8 (31.0%) were BFP
Glatt et al, 1991 [73]	Case series	n = 7 IVDUs (6 HIV+)	BFP: FTA	Illustrates that a high-titer false positive VDRL is possible. All patients had a titer ≥1:16
False positives: pregnancy				
Harrison et al, 1976 [74]	Retrospective study	n = 200 syphilitic sera of various stages and treatment; n = 500 sera from antenatal patients	BFP: FTA-ABS and TPHA Comparator: RPR and VDRL	The RPR was more sensitive than the VDRL (174/200 vs 167/200, respectively) The RPR was more specific than VDRL (1 FP in 500 vs 2 FP in 500, respectively)
el-Zaatari et al, 1994 [61]	Cross-sectional	n = 4328 sera tested by RPR and then rechecked with serial 2-fold dilutions up to 16-fold. n = 2065 pregnant women n = 1439 nonpregnant women n = 824 men	BFP: FTA-ABS	RPR: Female nonpregnant BFP: 1.1% Female pregnant BFP: .6% Male BFP: 1.3%
Smikle et al, 1990 [72]	Cross-sectional	n = 19 067 sera screened with VDRL. n = 441 general pop, n = 145 pregnant women VDRL + with a titer <1:8 were confirmed with FTA	BFP: VDRL <1:8 with a negative FTA	General population: 94/347 + VDRL with titer <1:8 (27%) were BFP. Pregnant women: 22/71 + VDRL with titer <1:8 (31.0%) were BFP.
False positive: autoimmune				
Dorwart and Myers, 1974 [75]	Retrospective cross-sectional	n = 74 with autoimmune connective tissue diseases, 41 also with SLE. Also, n = 19 healthy blood donors and n = 23 with syphilis	BFP: FTA-ABS	Connective tissue disease: RPR: 7/74 BFP VDRL: 6/74 BFP Blood donors: RPR: 0/19 BFP VDRL: 0/19 BFP 23/23 with syphilis were RPR and VDRL+
False positives: leprosy and yaws				
Achimastos et al, 1970 [76]	Cross-sectional	n = 50 leprosy patients without a self-reported history of syphilis tested with RPR circle card, the Kahn (a nontreponemal test no longer in use) and the FTA.	BFP: FTA	14/50 reactive by RPR card 13/50 reactive by Kahn None reactive by FTA—all considered BFP.
Garner, 1970 [77]	Cross-sectional retrospective	n = 270 patients with lepromatous leprosy	BFP: FTA-ABS	25/270 samples tested were positive by a nontreponemal test (not clear whether VDRL or RPR) and negative by FTA. 15/270 were positive by both treponemal and nontreponemal tests.
Chi et al, 2015 [78]	Cross-sectional	n = 155 children (<15 years old) with yaws. 24 were positive for *T. pallidum pertenue*	BFP: RT-PCR to detect *T. pallidum*, *T. pallidum pertenue* (yaws) and *T. pallidum endemicum* (bejel) in skin lesions	23/24 positive for *T. pallidum pertenue* PCR were RPR + (all were TPPA + as well) Overall 55/155 were positive for RPR.
False positive: HIV and HBV				
Hernández-Aguado et al, 1998 [79]	Prospective study	n = 5532 IVDU and n = 820 gay men.	BFP: FTA-ABS or TPHA	RPR or VDRL: HIV: 10.7% BFP vs 4.2% HCV: 4.5% vs 3.8% HBV: 8.3% vs 3.7% HIV and HBV were both statistically associated with an increased risk of BFP Of the 229 (12.3%) IVDUs who had BFPs at their first visit, only 47 of those 229 (20.5%) yielded a BFP result again at the subsequent visit a median of 18 months later
Rompalo et al, 1992 [80]	Cross-sectional	n = 4863 sera from patients attending an STD clinic	BFP: FTA	RPR: 6/159 BFP (4%) HIV+ 34/4387 BFP (.8%) HIV− (odds ratio, 5.0; 95% CI, 1.9–12.7).
Augenbraun et al, 1994 [81]	Retrospective	n = 156 women living with HIV vs n = 633 HIV- women in WIHS cohort	BFP: MHA TP and FTA-ABS Comparator test: RPR	6.9% and .2% of HIV-seropositive and HIV-seronegative women, respectively, had BFPs (*P* < .001; odds ratio, 39.45; 95% CI, 6.4–879.0). An association was found between injection drug use and BFPs for the population living with HIV
Geusau et al, 2005 [68]	Retrospective	n = 300 000 sera with age, sex, and stage of disease.	BFP: TPHA	VDRL+: 2799 patients (.92%) of the study population were positive, of whom 736 (26%) were BFP. BFP reactivity was found in .24% and was higher in women than in men (.27% versus .20%, respectively; *P* < .001) and in patients over 60 years of age (.34%) as compared with those under 60 (.25%; *P* < .001). People living with HIV (n = 1415) had a 10-fold higher rate of BFP tests (2.1% versus .24)
False positive: malaria				
Maves et al, 2014 [82]	Case-control cross-sectional	n = 73 with *Plasmodium vivax* malaria and n = 76 controls with other febrile illnesses.	BFP: TPHA	RPR: 8.2% (6/73) of patients with malaria due to *Plasmodium vivax* BFP. Range was up to 1:16; 0% BFP in controls.
False positive: HCV				
Thomas et al, 1994 [83]	Retrospective cross-sectional	n = 2672 patients attending an STD clinic	BFP: FTA-ABS	RPR: 9/330 (2.7%) of HCV Ab positive patients had a BFP, and 14/2154 (0.6%) HCV Ab negative patients had a BFP, P = .0017.
Sonmez et al, 1997 [84]	Cross-sectional	n = 21 syphilitic patients n = 50 HCV + patients n = 50 “healthy controls”	BFP: MHA-TP	VDRL: 10% (5/50) of patients with HCV had a BFP vs 0% BFP in controls.
False positives: drug				
Tuffanelli, 1968 [85]	Retrospective study	n = 54 former narcotic abusers n = 29 with history of BFP	BFP: FTA-ABS	VDRL: 18/54 repeatedly false positive. Average duration of BFP was 25 months, maximum BFP VDRL titer 1:64
Cushman and Sherman, 1974 [86]	Cross-sectional	n = 69 patients from methadone maintenance clinic tested initially and then retested at a mean of 23 +/− 7 months during methadone treatment. Controls Normal: n = 875 blood donors to blood bank	BFP: FTA	VDRL: Drug use group: 16/69 (23%) were BFP initially. 4/69 (6%) had BFP during treatment with methadone. Control group: 6/875 (.7%) were BFP, *P* < .001
False positive: vaccine				
Schueller and Izuno, 1976 [87]	Prospective study	n = 263 with VDRL changes following influenza, meningococcal, adenovirus, smallpox, tetanus, polio, and typhoid vaccines	BFP: FTA and clinical exam	VDRL: 1 of 263 healthy young recruits developed a BFP after vaccination. It reverted to nonreactive 6 months later.
Grossman and Peery, 1969 [88]	Prospective study	n = 575 patients without a history of prior syphilis who got a small pox vaccine.	BFP (1): serologic tests prior to vaccination were negative; (2) there was no evidence of recent syphilis; and (3) serologic reactions reverted spontaneously from positive to negative while under observation, or confirmatory tests (Reiter’s complement fixation) for syphilis gave negative results.	RPR: 10/575 BFP. All of these then either had a subsequent negative RPR or negative confirmatory test by Reiter’s complement fixation test.
Nonspecific syphilis stage				
Harrison et al, 1976 [74]	Retrospective study	n = 200 syphilitic sera of various stages and treatment and n = 500 sera from antenatal patients	FTA-ABS and TPHA	The RPR was more sensitive than the VDRL (174/200 vs 167/200, respectively) The RPR was more specific (1 FP in 500 vs 2 FP in 500, respectively)
Wilkinson et al, 1972 [89]	Cross-sectional	n = 1922 patients attending VD clinic	FTA-ABS	Sensitivity: VDRL+: 36/107 = 33.6% RPR+: 44/107 = 41.1%
Sharma et al, 1977 [90]	Cross-sectional	n = 50 “suspected to be suffering from syphilis”	FTA	Sensitivity: VDRL+: 36/42 = 89.0% RPR tear drop card+: 40/42 = 95.2%
Angue et al, 2005 [91]	Cross-sectional	n = 2100 women attending antenatal	VDRL comparator tests: Abbot Determine and Abbot Syfacard- R (RPR card test)	RPR+: sensitivity 56.3%; specificity 96.5% Determine: sensitivity 92.0%; specificity 94.6%
Fowler et al, 1976 [92]	Cross-sectional	n = 6488 sera from STD clinics:	FTA and RPCF	Sensitivity: RPR+ = 87.8% VDRL+ = 89.8% The VDRL was the least specific test compared to RPR but we could not calculate an exact value.
Stevens et al, 1978 [93]	Cross-sectional	n = 2300 sera	FTA-ABS	Sensitivity: RPR+ = 71.9% VDRL+ = 64.6%
Malm et al, 2015 [94]	Cross-sectional	n = 595 sera	Macro-Vue RPR card test	Sensitivity: VDRL+ = 76.1% (70.0–82.2) Specificity: VDRL+ = 93.0% (90.8–95.2)
Automated serologic tests				
Cate et al, 1971 [95]	Retrospective cross-sectional	n = 139 sera reactive by FTA-ABS n = 315 sera non reactive by FTA-ABS	For late or latent syphilis: FTA-ABS + some clinical (not well defined) available for 90% of patients Specificity: FTA negative	Sensitivity for “late or latent syphilis”: VDRL+: 102/139 = 73% Automated reagin using VDRL+: 98/139 = 71% General specificity: VDRL: 303/315 = 96% Automated reagin VDRL−: 305/315 = 97% There was a tendency for the automated reagin titer to be a dilution higher than that of the VDRL, but no statistical test to quantify this.
Lee et al, 2014 [96]	Cross-sectional	n = 112 serum samples (59 TPPA positive and 53 TPPA negative)	TPPA, some clinical information not well defined.	Sensitivity: Auto RPR (HiSense)+= 52.5% RPR Card+= 86.4% Specificity: Auto RPR = 94.3% RPR card = 94.3%. In 23 patients with treated syphilis, the automated RPR test showed earlier seroconversion 43.5% (10/23) than nonauto 4.3% (1/23)
Yukimasa et al, 2015 [97]	Cross-sectional	n = 1309 serum specimens: 77 “infective syphilis,” 153 “previous syphilis,” and 1079 “nonsyphilis”	FTA, and for the infective and previous syphilis cases, additional data from “clinical charts.”	Automated RPR Sensitivity in “infective syphilis” = 100% (77/77) Sensitivity in “previous syphilis” = 30.7% (47/153) Specificity in “nonsyphilis” = 71.8% (61/85) RPR card test Sensitivity in “infective syphilis” = 100% (77/77) Sensitivity in “previous syphilis” = 56.9% (87/153) Specificity in “nonsyphilis” = 74.1% (63/85) VDRL Sensitivity in “infective syphilis” = 100% (77/77) Sensitivity in “previous syphilis” = 65.4% (100/153) Specificity in “nonsyphilis” = 49.4% (42/85)
Stevens and Stroebel, 1970 [98]	Cross-sectional	n = 4441 serum samples.	VDRL slide test	Sensitivity: Automated reagin test+: 165/229 = 72.1% Specificity: Automated reagin test: 4205/4212 = 99.8%
Wilkinson et al, 1972 [89]	Cross-sectional	n = 1922 patients attending VD clinic	FTA-ABS	Sensitivity: VDRL+= 36/107 (33.6%) ART+= 44/107 (41.1%)
Mcgrew et al, 1968 [28]	Cross-sectional	n = 900 sera from “clinically defined” donor groups.	Overall: “clinically defined” without further details “False positive”: Reactive reagin test with “no clinical evidence or history of syphilis.”	Sensitivity compared with “clinical diagnosis” Syphilis: “treated and untreated” (n = 328), ART: 86.5%, VDRL slide: 88.4%, RPR card 86.9% Primary (n = 114) ART: 75.4%, VDRL slide 78.1%, RPR card 75.4% Secondary (*n* = 106) ART: 93.3, VDRL slide: 92.4%, RPR card: 94.3% Latent (n = 80) ART: 95.0%, VDRL slide: 98.8%, RPR card: 93.8% Late (n = 19): ART: 84.2%, VDRL slide: 84.2%, RPR card: 84.2% Congenital (n = 9): ART: 77.8%, VDRL slide: 88.9%, RPR card: 88.9% Percent positive: Presumed normal (n = 500): ART: .4%, VDRL slide: 3.6%, RPR card 5.2% False positive reactors (n = 101): ART: 85.2%, VDRL slide: 95.1%, RPR card: 83.2% Diseases other than syphilis: (n = 61): ART: 0, VDRL: 3.3%, RPR: 0.
Unpublished FDA data on FDA-approved automated RPR tests				
AIX 1000 RPR automated test system, 2015 [99]	Cross-sectional. No titer results reported for the AIX1000	Several different study populations	Comparator: ASI RPR card. Additionally, for the University of Washington clinically characterized samples, gold standard: Primary syphilis: genital lesion, + DF, and reactive treponemal test Secondary syphilis: rash or mucous patches or condyloma lata with reactive treponemal test Latent syphilis reactive treponemal and nontreponemal test with a nonreactive nontreponemal test for more than a year or unknown duration.	*n* = 765 prospectively collected sera, PPA: 95.5% (77.2–99.9), PNA: 99.9% (99.3–100). *n* = 2246 retrospectively collected sera from patients referred for syphilis testing: PPA: 97.2% (95.5–98.4), PNA: 99.1% (98.5–99.5%) Samples from people living with HIV, n = 250 nontreponemal test negative, n = 30 nontreponemal test positive PPA: 100% (90.5–100) PNA: 100% (98.8–100) University of Washington samples: All samples positive on AIX1000 and comparator, so 100% sensitive at all stages. Primary treated (n = 13): 100% agreement (79.4–100) Primary untreated (n = 12): 100% agreement (77.9–100) Secondary treated (*n* = 25): 100% agreement (88.7–100) Secondary untreated (n = 25): 100% agreement (88.7–100) Latent treated (n = 25): 100% agreement (88.7–100) Latent untreated (n = 25): 100% agreement (88.7–100)
Bio-rad laboratories Bioplex 2200 Syphilis Total and RPR Kit, 2017 [100]	Cross-sectional There is an option for + RPR titer result, titers can go to 1:64.	Multiple study populations	Comparator: an FDA-approved RPR test For clinically characterized samples, additionally, gold standard: diagnosis of stage of disease “made by a licensed physician based on the patient’s clinical symptoms, medical history and laboratory test results at the time of diagnosis”	*n* = 1001 prospectively collected samples without further clinical characterization PPA: 81.52% (72.39–88.13) NPA: 96.48% (95.07–97.50) n = 546 retrospective sera, n = 412 RPR or treponemal test positive samples test with the FDA-approved RPR or the Bioplex: PPA: 98.14% (96.37–99.05) NPA: 80.70% (72.51–86.90) n = 372 samples from pregnant women were tested with the FDA-approved RPR or the Bioplex: PPA: 100% (86.68–100) NPA: 98.27% (96.28–99.21) Population living with HIV: n = 362 samples from people living with HIV were tested with the FDA-approved RPR or the Bioplex; PPA: 85.71% (72.16–93.29) NPA: 90.63% (86.9–93.35) Serum from “apparently healthy individuals” n = 301 samples tested with the FDA-approved RPR or the Bioplex: PPA: 0% (0–48.98) NPA: 97.98% (95.66–99.07) Clinically characterized: Sensitivity: Primary untreated: Bioplex: 92.3% (24/26) RPR: 88.5% (23/26) Primary treated: Bioplex: 65.5% (19/29) RPR: 75.9% (22/29) Secondary untreated: Bioplex: 100% (25/25) RPR: 100% (25/25) Secondary treated: Bioplex: 88.5%(23/26) RPR: 80.8%(21/26) Latent untreated: Bioplex: 95.7% (22/23) RPR: 95.7% (22/23) Latent treated: Bioplex: 66.7% (18/27) RPR: 66.7% (18/27) All phases untreated: Bioplex: 95.9% (71/74) RPR: 95.0% (70/74) All phases treated: Bioplex: 73.2%(60/82) RPR: 74.4% (61/82)

HIV: 10.7% BFP in HIV+ vs 4.2% in HIV−. HCV: 4.5% BFP in HCV+ vs 3.8% in HCV−. HBV: 8.3% BFP in HBV+ vs 3.7% in HBV−.

Abbreviations: −, negative test result; +, positive test result; Ab, antibody; ANS, asymptomatic neurosyphilis; ART, antiretroviral therapy; BFP, biological false positive; CBFP, classical biological false positive; CI, confidence interval; CIA, chemiluminescence immunoassay; CSF, cerebrospinal fluid; CSF-RPR, rapid plasma reagin performed on the CSF modified to be similar to the CSF-VDRL method; DF, dark field; EDTA, ethylenediamine tetraacetic acid; e/o, evidence of; FDA, Food and Drug Administration; FP, false positive; FTA-ABS, fluorescent treponemal antibody absorption test; GUD, genital ulcer disease; *Haemophilus ducreyi*, *H. ducreyi*; HBV, hepatitis B virus; HCV, hepatitis C virus; HIV, human immunodeficiency virus; HSV, herpes simplex virus; ID, infectious disease; MHA-TP, microhemagglutination assay for *Treponema pallidum*; IVDUs, intravenous drug users; LP, lumbar puncture; MRI, magnetic resonance imaging; NS, neurosyphilis; N/A, not applicable; NPA, percent negative agreement; PCR, polymerase chain reaction; PNA, percent negative agreement; PPA, percent positive agreement; RPCF, Reiter protein complement fixation test; RPR, rapid plasma reagin; RPR-US, rapid plasma reagin test conducted on unheated serum; RST, reagin screening test; SNHL, sensorineural hearing loss; SNS, symptomatic neurosyphilis; STD, sexually transmitted disease; sx, symptoms; *T. pallidum*, *Treponema pallidum*; TPA, *Treponema pallidum* antibody; TPI, *Treponema pallidum* immobilization test; TPPA, *Treponema pallidum* particle agglutination; TPHA, *Treponema pallidum* haemagglutination test; TRUST, toluidine red unheated serum test; USR, unheated serum reagin; VD, venereal disease; VDRL, venereal disease research laboratory; WBC, white blood cells.

**Table 2.** Summary: Nontreponemal Antibodies for Various Stages of Syphilis

Stage of Syphilis	Nontreponemal Test	Sensitivity	Specificity	Quality of Studies
Primary	Serum RPR	62.5–76.1%^a^	N/A	High
	Serum VDRL	62.5–78.4%^b^	N/A	High
Secondary	Serum RPR	100%^c^	N/A	High
	Serum VDRL	100%	N/A	High
Early latent	Serum VDRL	85–100%	N/A	High
Late latent or unknown duration	Serum RPR	61%	N/A	High (1 study)
	Serum VDRL	64%	N/A	High (1 study)
Tertiary	Serum VDRL	47–64%	N/A	Lower (2 studies)
Neurosyphilis	CSF RPR	51.5–81.8%	81.8–100%	High
	CSF VDRL	49–87.5%	74–100%	High
Ocular	CSF VDRL	0–50%	N/A	Lower
Otic	CSF VDRL	5.5–5.6%	N/A	Lower (2 studies)

For full references, see the text of the paper and Table 1.

Abbreviations: CSF, cerebrospinal fluid; RPR, rapid plasma reagin; VDRL, venereal disease research laboratory.

^a^1 high-quality paper reported a sensitivity of 92.7%.

^b^1 high-quality paper reported a sensitivity of 50%.

^c^1 high-quality paper reported a sensitivity of 97.2%.

#### Conclusions

Serum RPR and VDRL are 62–78% sensitive for the diagnosis of primary syphilis.

### Secondary Syphilis

For secondary syphilis, we identified 11 high-quality papers with a clearly defined gold standard (see Tables 1 and 2) [2, 4, 6–9, 12, 16–18, 20] and 9 lower-quality papers with a less well-defined gold standard [23, 25–31, 101]. There was some heterogeneity in gold-standard definitions within the high-quality papers. Gold standards for the 11 high-quality papers included a clinical diagnosis, a clinical diagnosis “with positive darkfield,” and a clinical diagnosis with reactive treponemal or alternative nontreponemal tests (e.g., RPR, VDRL, or reagin screening test). Based on high-quality papers, the sensitivity of the VDRL was 100%. RPR was also reported as 100% sensitive, with the exception of 1 high-quality paper that reported a sensitivity of 97.2% [8]. No studies reported on specificity in the setting of secondary syphilis.

#### Conclusions

Serum RPR and VDRL are 97–100% sensitive for the diagnosis of secondary syphilis.

### Early Latent Syphilis

We identified 4 papers on early latent syphilis, and 2 were deemed high-quality based on having a well-defined gold standard [17, 20] (see Tables 1 and 2). The 2 others with less well-defined gold standards [23, 27] were deemed to be of lower quality. Based on these 4 studies, the overall sensitivity of VDRL ranged from 82.1–100%. Based on the 2 high-quality studies, the sensitivity of VDRL ranged from 85–100% [17, 20].

#### Conclusions

Based on a small number of studies (2 high-quality and 2 lower-quality studies), the sensitivity of VDRL for early latent syphilis ranges from 82–100% overall, and ranges from 85–100% based on the 2 high-quality papers.

### Late Latent Syphilis or Syphilis of Unknown Duration

We identified 2 high-quality papers with a clearly defined gold standard [17, 21] (see Tables 1 and 2) and 2 lower-quality papers with a less well-defined gold standard for late latent syphilis or syphilis of an unknown duration (see Table 1) [23, 27]. There was 1 high-quality study that reported a sensitivity of 61% for RPR [21]. The other 3 studies reported a sensitivity of 64–75% for VDRL [17, 23, 27] and the other high-quality study reported a sensitivity of 64% for VDRL [17].

#### Conclusions

Based on a small number of studies (2 high-quality and 2 lower-quality studies), the sensitivity of RPR and VDRL for diagnosing late latent syphilis ranges from 64–75% overall, and ranges from 61–64% based on the 2 high-quality papers.

### Tertiary Syphilis

Only 2 lower-quality papers, for which the gold standard was not well defined, were identified for tertiary syphilis (see Tables 1 and 2) [27, 31]. The first, based on 17 patients, reported a VDRL sensitivity of 47% [27]. The second, based on 58 patients, reported a VDRL sensitivity of 64% [31].

#### Conclusions

Based on 2 studies with very limited data, the sensitivity of serum VDRL for tertiary syphilis is 47–64%.

### Unspecified Syphilis Stage

We identified 12 high-quality studies [16, 74, 89–94, 101–104] with a well-defined gold standard and 8 [28, 30, 95, 105–109] lower-quality studies with a less well-defined gold standard, all of which provided little or no information on the syphilis stage. Without information on the syphilis stage, these papers were more difficult to interpret. However, the papers which contained data on both RPR and VDRL provided some useful information. There were 5 high-quality papers that reported a similar or higher sensitivity of the serum RPR as compared to the serum VDRL, independent of syphilis stage [74, 89, 90, 92–94], and 1 study that reported a lower sensitivity of RPR as compared with VDRL [91]. We found, 3 high-quality studies reported a higher specificity of RPR as compared to VDRL [74, 92, 94], while 1 high-quality study reported a slightly lower specificity of RPR as compared to VDRL [91]. Importantly, the serum RPR and VDRL titers were not equivalent (in 1 study, only 29% of sera had concordant titers), suggesting that serum RPR and VDRL titers should not be used interchangeably to manage patients [93] (see Table 1).

#### Conclusions

In general, serum RPR appears to be more sensitive than serum VDRL at detecting nontreponemal antibodies, independent of the syphilis stage. Based on more limited data, RPR also appears to be more specific than VDRL. Serum RPR and VDRL titers should not be used interchangeably to manage patients.

### Neurosyphilis

A neurosyphilis diagnosis is challenging, as no single laboratory test is perfectly sensitive and specific for a diagnosis. Additionally, clinical guidelines focus on obtaining lumbar punctures primarily in situations in which patients are symptomatic, as the significance of CSF abnormalities in the absence of symptoms is unclear. Assessing the literature was difficult as well. Definitions for neurosyphilis differed between studies. Additionally, some definitions were circular (for example, CSF VDRL was often included as part of the definition of neurosyphilis in studies reporting on the sensitivity of CSF VDRL), which complicates an interpretation of nontreponemal test performance characteristics. A mixture of symptomatic and asymptomatic patients was included in many studies, and it was not always possible to determine nontreponemal test characteristics separately for these groups. We identified 4 high-quality studies with a gold standard that did not include a CSF nontreponemal test [32–35] (see Tables 1 and 2); 13 moderate-quality studies where the gold-standard definition was partially circular (ie, included some element of the nontreponemal test itself) [17, 40, 110–120]; and 2 lower-quality studies with a less well-defined gold standard [121, 122]. We identified 2 high-quality case reports [36, 38] (and 2 lower-quality reports [123, 124]) and 1 high-quality study reporting on the amount of CSF blood contamination required to show a false-positive CSF VDRL [37] (see Table 1).

Based on the 4 high-quality studies, the sensitivity of the CSF VDRL ranged from 49–87.5% and the specificity ranged from 74–100% for diagnosing neurosyphilis [32–35]. In the studies in which the specificity was <90%, the gold standard was either CSF PCR for *T. pallidum*, symptoms (defined as new vision or hearing loss), clinical signs and symptoms with a positive CSF *Treponema pallidum* particle agglutination (TPPA) assay, or CSF white blood cells ≥ 10 and a positive CSF TPPA assay [33–35]. The gold standard for the study showing 99% specificity was positive serologies, reactive CSF fluorescent treponemal antibody (FTA), increased CSF protein >45 mg/dL, and CSF pleocytosis ≥10 cells/mm^3^ [32]. The sensitivity of CSF RPR ranged from 51.5–81.8% and the specificity ranged from 81.8–100% [32, 34, 35]. For symptomatic neurosyphilis, the sensitivity of CSF VDRL ranged from 66.7–87.5% and the specificity ranged from 78.2–90.2% [32–35]. For symptomatic neurosyphilis, the sensitivity of CSF RPR ranged from 51.5–100% and the specificity ranged from 89.7–90.2% [32, 34, 35]. However case definitions for symptomatic neurosyphilis had significant heterogeneity (2 studies by Marra et al [33, 34] defined symptoms as “vision or hearing loss”; for the remaining studies, symptoms were not further defined). There was 1 high-quality study that reported a sensitivity of 76.2% and specificity of 93.1% for CSF TRUST [35]. Limited data suggest that the sensitivity of CSF RPR may be lower than that of CSF VDRL [34]. There were 2 good-quality case reports describing false positive CSF VDRL results in the setting of central nervous system malignancy [36, 38]. Finally, 1 paper reported on a laboratory experiment in which syphilitic blood was added to CSF, demonstrating the principle that bloody contamination of the CSF during a traumatic tap could lead to a false-positive CSF VDRL in a patient with syphilis, particularly a patient with a high VDRL serum titer [37]. (For a discussion of the use of CSF treponemal antibodies in neurosyphilis, please see Park et al in this issue.) Studies have suggested the neurosyphilis risk is highest in persons living with HIV with a serum RPR of >1:32 [125–127].

#### Conclusions

A neurosyphilis diagnosis is challenging. Different definitions for neurosyphilis across studies, the heterogeneity of gold standards used, and the inclusion of a mixture of symptomatic and asymptomatic patients were highlighted as limitations. Based on current data, there is no recommendation for the use of 1 assay over the other in the laboratory diagnosis of neurosyphilis, though limited data suggest that CSF RPR may be less sensitive than CSF VDRL. CSF VDRL is 49–87% sensitive and 74–100% specific for diagnosing neurosyphilis. CSF RPR is 51–82% sensitive and 82–100% specific for diagnosing neurosyphilis. Due to significant heterogeneity in case definitions, a lack of gold standards, and the wide range of results, it is not possible to give definitive information on the performance characteristics of CSF nontreponemal tests in neurosyphilis diagnosis.

### Ocular Syphilis

The diagnosis of ocular syphilis is heavily reliant on a clinical assessment in the setting of reactive serum serologies, as the CSF may be completely normal in up to 40% of patients. Furthermore, there is not a particular pathognomonic sign or symptom of ocular syphilis. We found 18 lower-quality studies [39–56] relating to ocular syphilis (see Tables 1 and 2). Most were small cases series with limited numbers. Based on these studies, the sensitivity of CSF VDRL ranged from 0–50%, with many studies reporting a sensitivity of ≤30% [39–56]. There were 5 studies that reported a sensitivity of 24–100% for serum VDRL [39, 42, 45, 50, 54] and 5 studies that reported a sensitivity of serum RPR of 85–92.3% [44, 46, 50, 52, 55].

#### Conclusions

The sensitivity of CSF VDRL in ocular syphilis is <50%.

### Otic Syphilis

Similar to ocular syphilis, the diagnosis of otic syphilis relies on a clinical assessment in the setting of reactive serum serologies, as over 90% of persons with otic syphilis may have normal CSF parameters. We identified 4 lower-quality papers, all cases series (see Table 1) [57–60]. Of these, 2 reported a sensitivity of CSF VDRL ranging from 5.4–5.6% [59, 60] (see Table 2). The sensitivity of serum VDRL was reported in 1 study at 22.2% [59], and the sensitivity of serum RPR was reported in 2 studies at 55–88.9% [57, 58].

#### Conclusions

Limited data suggest that the sensitivity of CSF VDRL is poor (<10%) in otosyphilis.

### False Negatives: Prozone Reaction

We identified 2 high-quality papers with a clear gold standard [61, 63] and 1 high-quality case report [62], as well as 2 lower-quality case reports [128, 129] (see Table 1). Based on the 2 high-quality papers, the prevalence of false-negative results from nontreponemal syphilis tests is rare (<0.85% of those tested) [61, 63] The prozone reaction generally refers to a false-negative response arising from cases in which high antibody titers interfere with the antigen-antibody lattice network formation necessary to visualize a positive flocculation test [57]. The prozone reaction can occur during any stage of syphilis but is more common in primary and secondary syphilis; neurosyphilis and pregnancy may increase the risk of the prozone reaction [61, 63]. There was 1 large study of 46 856 samples that reported that a third of prozone reactions may occur when titers are ≤1:16 [63]. Finally, 1 study reported that a cold centrifuge led to false negatives [62]. There was no formal guidance on the optimal number of serial dilutions when investigating a possible prozone reaction. In 1 study, titers were rechecked with serial dilutions up to 16-fold [61]; in the other, they were diluted from 1:1 to 1:32 [63].

#### Conclusions

The prevalence of false-negative results from nontreponemal syphilis tests is rare (<0.85% of those tested.) The prozone phenomenon can occur during any stage of syphilis but is more common in primary and secondary syphilis; neurosyphilis and pregnancy may increase the risk of the prozone reaction. A third of prozone reactions may occur when titers are ≤1:16. False negatives may be more common if sera are centrifuged at colder temperatures (eg, 4℃ vs 27℃).

### Causes of Reactive Nontreponemal Tests Other than Syphilis

We identified 51 papers [12, 14, 16, 27, 28, 61, 64–88, 91, 106, 108, 130–146] that reported on causes of reactive nontreponemal tests other than syphilis—that is, biological false positives (BFPs)—with some focusing on positives in specific populations or conditions (such as pregnancy, old age, leprosy, illicit drug use, malaria, HIV, hepatitis C virus, other treponemal diseases, autoimmune diseases, and vaccines; see Tables 1 and 3). The studies were of mixed quality: many were small, and comparator groups were often lacking. In most large studies with a well-defined gold standard (generally a negative treponemal test), the overall prevalence of BFPs in those general populations tested for syphilis was ≤1.5% [14, 64–69]. Several factors were associated with BFPs, including older age, certain autoimmune diseases, leprosy, yaws, and HIV [68, 71, 75–81]. More limited data exist on the associations of malaria, hepatitis B infection, hepatitis C infection, and injecting drugs with increased risks of BFPs [79, 82–86]. The data for the association of BFPs and pregnancy are conflicting [61, 72, 74]. The data do not suggest a significant impact of vaccination on BFPs [87, 88]. In general, BFPs are more likely to occur when the nontreponemal titers are low, usually ≤1:8 [69, 72] (but there is clear documentation of exceptions where BFPs occur with higher nontreponemal titers; in general, those titers were usually ≤1:64) [73, 82, 85]. The duration of BFPs may depend on their underlying etiology. BFPs, in most cases, tend to revert to nonreactive. A small study suggests that the majority (83.3%) serorevert within approximately 10 weeks [70]. In another small study of patients with narcotics addictions, the average duration of the BFP was 25 months [85].

**Table 3.** Causes of Positive Nontreponemal Tests Other Than Syphilis

Cause	Quality of Data
Yaws	High
Leprosy	High
Autoimmune conditions	High
Human immunodeficiency virus	High
Hepatitis B virus	Lower
Hepatitis C virus	Lower
Malaria	Lower
Drug use (narcotic, methadone)	Mixed

For full references, see the text of the paper and Table 1. Data on pregnancy are conflicting.

#### Conclusions

In most large studies with a well-defined gold standard (generally a reactive treponemal test), the overall prevalence of BFPs in the general populations tested for syphilis was ≤1.5%. Several factors were associated with BFPs, including older age, certain autoimmune diseases, leprosy, yaws, and HIV. More limited data exist on the associations of malaria, hepatitis B infection, hepatitis C infection, and injecting drugs with increased risks of BFPs. The data for the association of BFPs and pregnancy are conflicting. The data do not suggest a significant impact of vaccination on BFPs. In general, BFPs tend to occur when the nontreponemal titers are low, but there is clear documentation of exceptions where BFPs occur with higher nontreponemal titers. The duration of BFPs may depend on their underlying etiology. BFPs, in most cases, tend to revert to nonreactive.

### Automated Nontreponemal Tests

There are limited data available comparing automated nontreponemal tests (platforms which allow for the automated qualitative and quantitative detection of nontreponemal antibodies in serum or plasma) and manual nontreponemal tests [28, 89, 95–98]. Only 2 automated assays are currently FDA approved (see Table 1) [99, 100]. Data vary, but studies suggest similar overall performances of automated and nonautomated tests. A large number of samples were tested in the unpublished data submitted to the FDA, although not all the sample testing was relevant. For 1 test where the comparator was an RPR card test, the overall agreement was close to 100%, with the percent positive agreement (PPA) ranging from 95–100% and the percent negative agreement ranging from 99.1–100% [99]. For the other, in which the comparator was “an FDA-approved RPR test,” the PPA ranged from 81.5–100% (with 1 instance, with only 6 samples where PPA was 0%) and the percent negative agreement ranged from 80.7–98% [100]. The automated tests may only provide a limited range of titer dilutions, beyond which manual procedures are necessary to achieve endpoint titration. Endpoint titers are necessary for patient management and should be obtained in all cases, without exception.

#### Conclusions

Based on limited data (including some unpublished manufacturer’s data submitted to the FDA), the automated nontreponemal tests appear to have reasonable performance as compared to the nonautomated tests. However, manual procedures may be necessary to achieve endpoint titration, which is critical for patient management.

## GAPS IN THE FIELD AND OVERALL CONCLUSIONS

Overall, there is a need to better define the performance characteristics of nontreponemal tests, particularly in neurosyphilis and the latent stages of syphilis, with clinically well-characterized samples, including in populations living with and without HIV. Published data are needed on FDA-approved, automated nontreponemal tests. Additionally, questions remain regarding whether titers from automated tests are interchangeable with manual tests, and what recommendations should be made regarding manual testing proficiency (especially as expertise declines with the introduction of the automated tests). Data are needed to better define the performance characteristics of nontreponemal tests in neurosyphilis. Studies which exclude the nontreponemal test in question as part of the gold-standard definition would be particularly valuable. Additional data are needed to gain a better understanding of the relationship between disease activity and nontreponemal antibody titers. Evidence-based guidelines for prozone titrations and improved criteria and diagnostics for neurosyphilis (as well as ocular and otic syphilis) are needed.

## Notes

***Acknowledgments.*** The authors thank the members of the Centers for Disease Control and Prevention Sexually Transmitted Disease Laboratory group and external experts who developed or reviewed the key questions or supported the systematic literature reviews, and our colleague, Dr John Papp, for his insightful review of this manuscript.

***Financial support.*** This work was supported by the Centers for Disease Control and Prevention (to S. K.). S. T. is supported by NIH grant K23AI125715 (PI: Tuddenham).

***Supplement sponsorship.*** This article appears as part of the supplement National Coalition of STD Directors, sponsored by the CDC.

***Potential conflicts of interest.*** S. S. K. is a federal employee at and has received personal fees from the US Centers for Disease Control and Prevention. S. T. has been a consultant for Biofire Diagnostics and Roche Molecular Diagnostics, and has received a speaker honorarium from Roche Molecular Diagnostics. K. G. G. reports no potential conflicts. All authors have submitted the ICMJE Form for Disclosure of Potential Conflicts of Interest. Conflicts that the editors consider relevant to the content of the manuscript have been disclosed.
